# Monitoring Herbivorous Fishes as Indicators of Coral Reef Resilience in American Samoa

**DOI:** 10.1371/journal.pone.0079604

**Published:** 2013-11-06

**Authors:** Adel Heenan, Ivor D. Williams

**Affiliations:** 1 Joint Institute of Marine and Atmospheric Research, University of Hawaii at Manoa, Honolulu, Hawaii, United States of America; 2 Coral Reef Ecosystem Division, Pacific Islands Fisheries Science Center, Honolulu, Hawaii, United States of America; Leibniz Center for Tropical Marine Ecology, Germany

## Abstract

Resilience-based management aims to promote or protect processes and species that underpin an ecosystem's capacity to withstand and recover from disturbance. The management of ecological processes is a developing field that requires reliable indicators that can be monitored over time. Herbivory is a key ecological process on coral reefs, and pooling herbivorous fishes into functional groups based on their feeding mode is increasingly used as it may quantify herbivory in ways that indicate resilience. Here we evaluate whether the biomass estimates of these herbivore functional groups are good predictors of reef benthic assemblages, using data from 240 sites from five island groups in American Samoa. Using an information theoretic approach, we assembled a candidate set of linear and nonlinear models to identify the relations between benthic cover and total herbivore and non-herbivore biomass and the biomass of the aforementioned functional groups. For each benthic substrate type considered (encrusting algae, fleshy macroalgae, hard coral and turf algae), the biomass of herbivorous fishes were important explanatory variables in predicting benthic cover, whereas biomass of all fishes combined generally was not. Also, in all four cases, variation in cover was best explained by the biomass of specific functional groups rather than by all herbivores combined. Specifically: 1) macroalgal and turf algal cover decreased with increasing biomass of ‘grazers/detritivores’; and 2) cover of encrusting algae increased with increasing biomass of ‘grazers/detritivores’ and browsers. Furthermore, hard coral cover increased with the biomass of large excavators/bio-eroders (made up of large-bodied parrotfishes). Collectively, these findings emphasize the link between herbivorous fishes and the benthic community and demonstrate support for the use of functional groups of herbivores as indicators for resilience-based monitoring.

## Introduction

Addressing both the local and global threats to ecosystems is a challenge for natural resource managers. The theory of ecological resilience provides a foundation to deal with these threats of disparate scale: ecosystems with increased resilience have greater capacity to recover from stress without switching into an alternate and often undesirable state [1], [2], [3]. Given that coral reefs face local impacts (e.g. intensive fishing and coastal development), along with global impacts (e.g. climate related coral bleaching), there has been a strong push toward managing coral reefs with the goal of maximizing resilience [4], [5], [6].

For coral reefs, an obvious component of ecological resilience is the ability to maintain or recover to a coral-dominated state following disturbance. Although shifts into various alternate states have been recorded [7], the most widely recognized and reported shift is from coral to macroalgal dominance [8]. Caged exclusion experiments have shown that in the absence of herbivores, fleshy macroalgae proliferate and coral recruitment and recovery following bleaching is suppressed [9], [10], [11], [12]. Following large-scale disturbances, such as storm damage and bleaching events which cause coral loss, subsequent shifts from coral to algal dominated states can occur, with depletion of herbivores often implicated in the inability of reefs to regain coral cover [13], [14]. Furthermore, reefs with higher abundance and/or biomass and diversity of herbivores tend to have lower macroalgal cover [15], [16], [17], [18], [19]. The prospect that herbivore-targeted management interventions might increase the resilience of reef systems underlies the appeal of resilience-based management as it suggests that local management may be able to locally mitigate some of the unpredictable effects of larger-scale stressors.

Although resilience is a useful concept, a gap nevertheless exists between resilience theory, experimental studies, and the quantitative information needed to support resilience-based management decisions. A necessary step towards improved capacity for resilience-based management is to improve the scope for quantifying and monitoring ecological processes that confer resilience. Using functional groups of species as operational indicators of resilience is one approach that can be used to recognize vulnerability before disturbance events [2]. Functional groups of herbivorous fishes, which group fishes by feeding mode, have been proposed as resilience indicators based on the impact they have on coral-algal dynamics.

The broadest distinction in the herbivore functional groups is between fishes which graze predominantly on algal turfs and those which browse on fleshy macroalgae. Grazers may prevent macroalgae from becoming established by feeding on diminutive macroalgae and turf algae [10], [20]. ‘Browsers’ have the potential to reverse a macroalgal phase shift as they can reduce the overgrowth and shading of coral by selectively feeding on macroalgae [21], [22]. Within the grazing group, a further distinction is made based on the amount of the underlying substrate that is removed during feeding. ‘Grazers/detritivores’ consume large amounts of algal turf while they brush the epithlic algal matrix for detritus [23], [24]. This group is distinct from scraping and excavating parrotfishes which scrape and take bites off the reef matrix and may open up new sites for calcifier settlement, thereby potentially promoting resilience by facilitating the settlement, survival and growth of crustose coralline algae and coral [25], [26]. The scraping and excavating parrotfish are further divided by size (‘scrapers/small excavators’ and ‘large excavators/bio-eroders’). Large excavators/bioeroders can act as major agents of bioerosion, consuming greater quantities of the reef matrix than their smaller counterparts. For this reason, the deep bites by large parrotfishes may be of increased functional importance in terms of retarding recovery of fleshy and turf algae and facilitating coral recruitment through the opening up new settlement sites [21].

The functional impact of these herbivore groups will likely vary with the intensity of feeding. For instance, herbivorous fish grazing tends to vary with depth and with coral cover, whereby locations with high coral cover have grazing concentrated on the rather smaller proportion of substrate occupied by algae [15], [27], [28]. Furthermore, herbivory is only one of several factors that influence coral-algal dynamics. For example, macroalgal cover increases with decreasing water quality, coral community composition is influenced by sedimentation load [29], [30], [31] and macroalgal cover is increased in areas exposed to wave impacts while the density of coral juveniles is higher in sheltered compared to exposed reefs [32]. There is, however, substantial evidence that herbivorous fish can and do mediate benthic algal cover [15], [17], [28], [33], [34].

For a resilience-based approach to be successfully integrated into ecosystem monitoring and management, resilience indicators have to be based on well-accepted scientific theory. The negative correlation between herbivore biomass and macroalgal cover is generally well supported, which suggests functional groups of herbivores could serve as indicators for increased vulnerability to macroalgal dominance following disturbance. There is, however considerably less evidence that supports the role of herbivore functional groups (large excavators/bio-eroders and scrapers/small excavators, parrotfishes in both cases) in facilitating the settlement, growth and survival of crustose coralline algae and coral [10], [21], [26]. Whether these excavating parrotfishes facilitate the settlement of corals by opening up space for settlement is uncertain, because they can also contribute to the mortality of recently settled corals through incidental grazing However, in most cases, the net benefit of bio-erosion caused by excavating parrotfishes is expected to outweigh the negative impact corallivory may have on corals at the population level [35].

The aim of this investigation was to assess how herbivorous fish biomass relates to benthic composition and cover. Using data collected from 240 sites in the five island groups of the American Samoan Archipelago, we used generalized additive mixed effects models to test how herbivorous fish biomass relates to benthic composition. Specifically, we assessed whether there was an increased power to predict benthic cover when the resilience indicator approach of pooling herbivores into functional groups was used compared to pooling all herbivores together and to total fish biomass. We found that the biomass of herbivorous fishes varies with benthic composition in ways one might predict based on their functional link to resilience.

## Methods

### Ethics statement

Permission to work in American Samoa was granted under permits from the National Park of American Samoa (NPSA-2010-SCI-0002), the Pacific Reefs National Wildlife Refuge (12521-10001), the Office of National Marine Sanctuaries (Fagatele Bay National Marine Sanctuary FBNMS-2010-001) and the Department of Marine and Wildlife resources (2010/01).

### Study location

**Figure 1:**
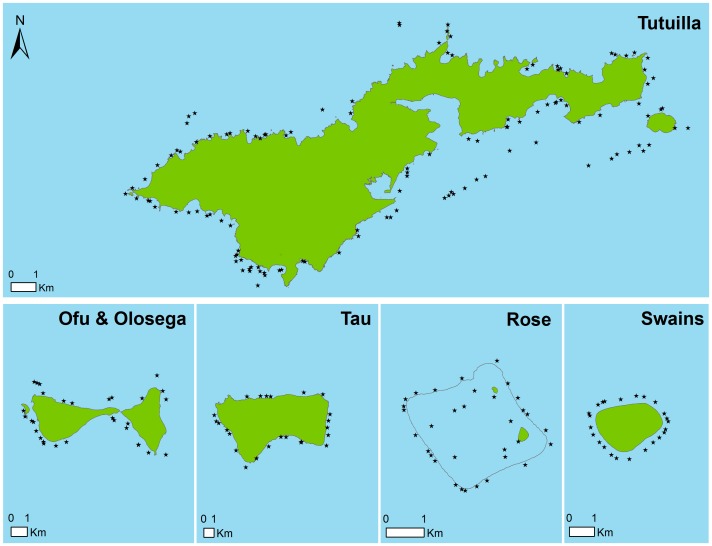
Map showing the location of the survey sites. As part of the NOAA Pacific Reef Assessment and Monitoring Program, 240 sites were selected using a random depth-stratified design over hardbottom substrate in depths of 1–30-m. At each site the fish community was surveyed by means of a stationary point count method and the benthic composition examined via a photo-transect.

The data used for this study was collected by the NOAA Coral Reef Ecosystem Division (CRED) of the Pacific Islands Fisheries Science Center (PIFSC) as part of the Pacific Reef Assessment and Monitoring Program (Pacific RAMP). In 2010, 240 coral reef sites in the American Samoan Archipelago were surveyed (Fig. 1). Sites were selected using a random, depth-stratified design encompassing all 1–30 m deep hard bottom habitats across the islands of Ofu and Olosega, Rose Atoll, Swains, Ta'ū, and Tutuila. The depth strata were shallow (<6-m), mid-depth (6–18-m) and deep (18–30-m).

### Quantifying herbivorous fish biomass

The fish assemblage was surveyed using a stationary point count method (SPC). The aim of this study was to apply the herbivorous fish resilience indicators to an existing reef monitoring program. The SPC is the method of choice in the Pacific RAMP because it allows multiple objectives for collecting data on fish assemblages to be met across a variety of survey locations (∼46 islands and atolls in the western central Pacific). At each site, pairs of divers laid a 30-m gray Dacron line along a depth contour. Divers took positions at 7.5-m and 22.5-m along the transect line and visualized themselves in the center of adjacent 15-m diameter cylinders. During an initial timed 5-min period, divers compiled a list of all fish species present within or passing through their cylinder. At the end of the 5-min period, divers systematically worked through the species list, recording the number and total length of all individuals of each species within that cylinder. To do this, divers maintained their position in the center of the cylinder and slowly rotated to perform a visual sweep of the cylinder area. During the counting and sizing period, divers grouped species with similar search image together (e.g. benthic associated butterflyfishes). Small, generally site-attached and semi-cryptic species such as small damselfishes and wrasses were counted last, at which point divers conducted a wider swim throughout the cylinder (see [36] for full method protocol). Biomass per fish was then calculated using length-weight relationships [37], [38]. Data from the two adjacent SPC surveys were pooled to create biomass estimates per site. All herbivorous fishes were categorized into the following functional groups; browsers, grazers/detritivores, large excavators/bio-eroders and scrapers/small excavators and the biomass (g m^−2^) of each of these functional groups calculated per site [21].

### Measuring benthic cover

On completion of the fish survey, 30 photographs were taken at 1-m intervals on the transect line. Divers used a 1-m length monopod to position a digital camera (Canon PowerShot SD1300IS, 12.1 megapixel) above the substrate, framing an area of approximately 0.7-m^2^. Benthic images were analyzed using CPCe version 4.1 [39]. Ten random points per image were selected and identified to the following benthic functional groups, encrusting algae (which includes encrusting macroalgae, crustose coralline algae (CCA) growing on hard substrate and CCA growing on rubble), hard coral, fleshy macroalgae, turf algae, sand and soft coral.

### Data modeling

We used general additive models (GAMs) to assess relations between benthic cover of each substrate type and the biomass of functional groups of herbivorous fishes. The percentage cover of each benthic category was modeled as the response and the biomass of fishes as the predictor variables within a mixed modeling framework. Separate sets of models were run per benthic category (encrusting algae, hard coral, fleshy macroalgae and turf algae) and the candidate models run for each benthic category included each combination of the herbivore functional groups, plus three reference models: the null model (one with no predictor variable), one with total herbivore fish biomass, and one with total fish biomass (with herbivorous and non-herbivorous fishes pooled together). All models were fitted as both generalized linear (GLMs) and GAMs, and a comparison of models indicated that in all cases of benthic substrate type, the non-linear models provided the best fit, which confirmed that GAMs were the appropriate model for benthic cover and fish biomass. Preliminary data inspection revealed that the biomass of scrapers/small excavators was collinear with the grazers/detritivores, therefore we did not run models which included both of those two groups. Fish biomass and depth were specified as fixed effects, with island as a random effect to account for the non-independence of the data points collected at the same islands [40].

For each benthic category, the Akaike Information Criterion (AIC) corrected for a small sample size (AICc) was used to evaluate the candidate models. The difference in AICc relative to the best model (dAICc) and an AICc-based relative importance weight (w_i_) for the candidate set of models (i) was used to identify the best fitting model by calculating the relative support for each model, the best models being those with the lowest AICc value. We report the top ranking models (any within 15% of the model-based support from the w_i_ results [41]), alongside the three reference models (null model, total herbivorous fish biomass, and total fish biomass). The AICc was also used to manually select the number of knots and so the degree of smoothing in the curve fitted for the GAMs. Model assumptions were validated through visual inspection of the residuals. In the case of fleshy macroalgal and turf algal cover, a departure from the assumption of homogeneity of variance was apparent; therefore, models were refit with a Gamma error structure. All models were fit in R (R Development Core Team 2011) using the mcgv (version: 1.7–5) and the lme4 (version: 0.999375-42) packages.

## Results

### Benthic composition

**Figure 2:**
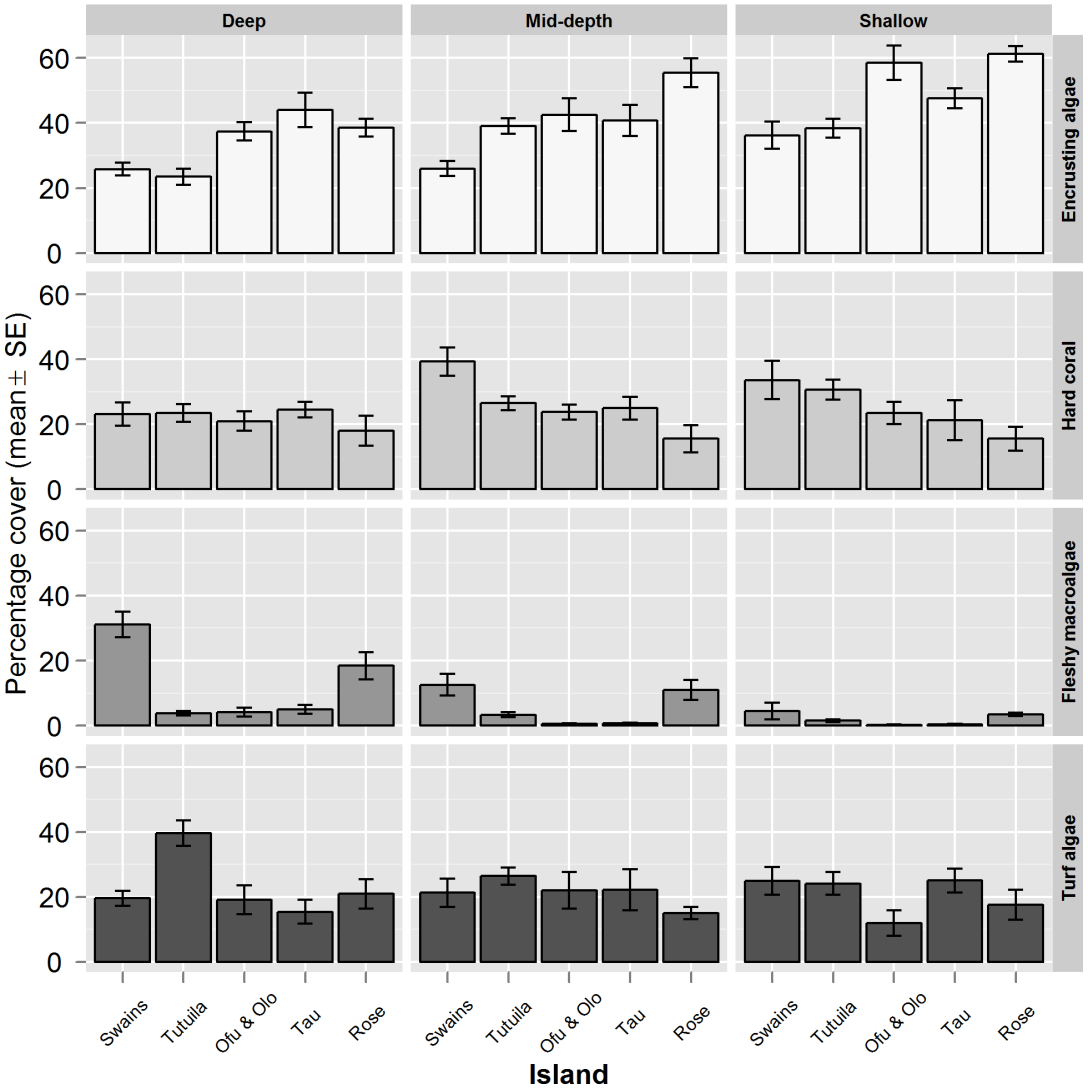
Variation in the benthic composition around islands in American Samoa by depth zone. The mean percentage cover (standard error (SE) bars) of encrusting macroalgae, hard coral, fleshy macroalgae and turf algae. Mean cover was calculated by pooling sites surveyed per depth zone per island. Depth zone ranges were shallow: 0–6-m, mid-depth: 6–18-m and deep: 18–30-m).

Mean encrusting algal cover was 37.9% (SE 1.2) at sites surveyed in American Samoa, however, this varied by island, with Rose Atoll and Ofu & Olosega having particularly high encrusting algal cover (Fig. 2). Fleshy macroalgal cover averaged 5% (SE 0.5) and tended to be lower at shallow sites across all islands (Fig. 2). Furthermore, Rose and Swains showed notably higher fleshy macroalgal cover, particularly in the mid to deep sites. Hard coral cover was broadly similar across all islands, with a mean cover of 25.1% (SE 0.9). However, Swains appeared to have higher coral cover at mid-depth and shallow sites. Mean cover of turf algae was 25.1% (SE 1.2) across the archipelago, with deep sites at Tutuila having notably higher cover than other islands and depth categories (39.5% (SE 3.9); Fig. 2).

### Herbivorous fish assemblages

**Figure 3:**
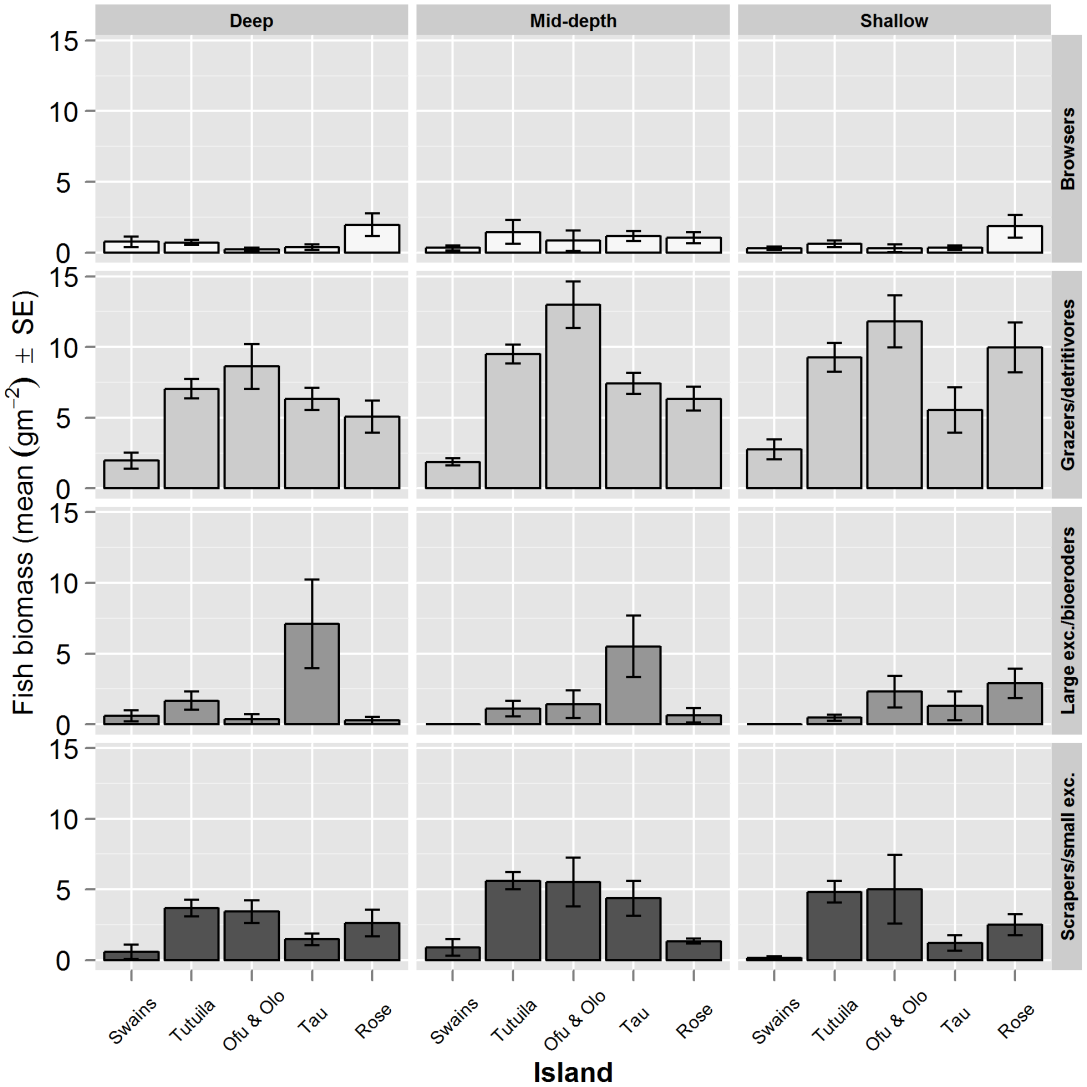
Variation in the biomass of functional groups of herbivorous reef fishes in American Samoa. The mean biomass (gm^−2^) (SE) of A) browsers, B) grazers/detritivores, C) large excavators/bio-eroders and D) scrapers/small excavators per island. Mean biomass per groups was calculated by pooling all sites surveyed per island. Ofu & Olo = Ofu & Olosega.

Total herbivore biomass was considerably lower at Swains (3.42 g m^−2^ (SE 0.70)) than at the other islands in American Samoa (Ofu & Olosega: 17.54 g m^−2^ (SE 1.76), Tutuilla: 15.20 g m^−2^ (SE 0.83), Ta'ū: 14.73 g m^−2^ (SE 1.85), Rose: 11.83 g m^−2^ (SE 1.47)). The biomass of browsers was notably low relative to other functional groups, and this was apparent across all islands, with browsers only constituting 5% of total herbivorous fish biomass (Fig. 3). Mean browser biomass was 0.71 g m^−2^ (SE 0.08) across the archipelago, with Rose Atoll having higher browser biomass compared to other islands (1.50 g m^−2^ (SE 0.38)). The dominant herbivore functional group was grazers/detritivores (57% of total biomass), followed by scrapers/small excavators (27%) and large excavators/bio-eroders (10%). Grazers/detritivore biomass averaged 7.86 g m^−2^ (SE 0.34) for all islands, with Swains having visibly lower biomass across all depth zones (overall mean grazer/detritivore biomass at Swains: 2.15 g m^−2^ (SE 0.30); Fig. 3). Archipelago-wide, the mean scraper and small excavator biomass was 3.78 g m^−2^ (SE 0.27). Scraper and small excavator biomass was broadly similar in Tutuila (4.77 g m^−2^ (SE 0.37)) and Ofu & Olosega (4.65 g m^−2^ (SE 0.97)), both of which were considerably greater than Rose (2.10 g m^−2^ (SE 0.39)), Ta'ū (2.48 g m^−2^ (SE 0.57)) and Swains (0.56 g m^−2^ (SE 0.27); Fig. 3). Finally, the mean biomass of large excavators/bio-eroders at sites surveyed in American Samoa was 1.47 g m^−2^ (SE 0.26). Notable island deviations from the archipelago mean were lower biomass recorded at Swains (0.22 g m^−2^ (SE 0.15)) and an increased biomass at Ta'ū (5.05 g m^−2^ (SE 1.47); Fig. 3).

### Relationship between benthic composition and herbivorous fishes

Overall, models which pooled herbivorous fish into functional groups were favored in the model selection process; for all categories of benthic cover, models with the greatest deviance explained were those with functional group biomass and depth as the predictor variables rather than total herbivore biomass and/or total fish biomass. Deviance explained by the best models ranged widely from between 9 and 50% (Table 1).

**Table 1:** Variance explained in benthic variables by herbivorous fish in American Samoa.

Benthic category	Model	Deviance explained (%)	AICc	edf	dAICc	w_i_
**Encrusting algae**	**G**	42	**1828**	**9.0**	**0**	**0.462**
	**B+G**	43.3	**1828.9**	**10.3**	**0.9**	**0.299**
	**G+L**	42.5	**1830.4**	**9.7**	**2.4**	**0.138**
	**All herbivores**	38.3	1844.7	8.9	16.7	<0.001
	All herbivores + all non-herbivores	38.3	1846.8	9.8	18.8	<0.001
	Null		1884.9	4.0	56.9	<0.001
**Hard coral**	**L**	8.95	**1854.1**	**6.9**	**0**	**0.241**
	**B+L**	9.34	**1855.4**	**7.9**	**1.2**	**0.130**
	**G+L**	8.99	**1855.8**	**7.7**	**1.7**	**0.104**
	**L+S**	8.95	**1856.2**	**7.8**	**2.1**	**0.085**
	**B**	7.93	**1856.5**	**6.8**	**2.4**	**0.072**
	**All herbivores + all non-herbivores**	7.92	**1856.6**	**6.8**	**2.5**	**0.070**
	**B+G+L**	9.45	**1856.9**	**8.7**	**2.8**	**0.060**
	**G**	7.45	**1857.2**	**6.6**	**3.1**	**0.052**
	Null		1860.5	4.0	6.4	0.010
**Fleshy macroalgae**	**G**	**50.4**	**1116.2**	**9.3**	**0**	**0.491**
	**G+L**	**51.2**	**1117.9**	**12.0**	**1.7**	**0.208**
	**B+G**	**50.8**	**1118.4**	**11.2**	**2.3**	**0.156**
	**B+G+L**	**51.5**	**1118.6**	**13.0**	**2.4**	**0.146**
	All herbivores	46.9	1132.4	8.9	16.2	<0.001
	All herbivores + all non herbivores	47.1	1134	10.2	17.9	<0.001
	Null		1528	4.0	411.8	<0.001
**Turf algae**	**G+L**	14.9	**1874.2**	**7.58170**	**0**	**0.293**
	**All herbivores**	14	**1874.7**	**6.63499**	**0.5**	**0.223**
	**G**	13.8	**1875.1**	**6.62265**	**0.9**	**0.188**
	**All herbivores + all non**	14.5	**1876.2**	**7.98924**	**2**	**0.107**
	Null		1987.6	4	113.4	<0.001

Model selection results showing the top-ranked models (in bold) of fish biomass and benthic cover in American Samoa. All models contained depth and the functional groups below as fixed effects, in addition to island as a random effect. Null and models with all herbivores and total fish biomass (all herbivore plus all non-herbivore biomass) are also reported. AICc =  Akaike information criterion corrected for small sample size, edf =  estimated degrees of freedom, dAICc =  the difference in AICc relative to the best model, w_i_ =  Akaike weight for the set of candidate models, B =  browsers, G =  grazers/detritivores, L =  large excavators/bio-eroders, S =  scrapers/small excavators, All herbivores  =  biomass of all herbivorous fishes, All non-herbivores  =  total fish biomass.

**Figure 4:**
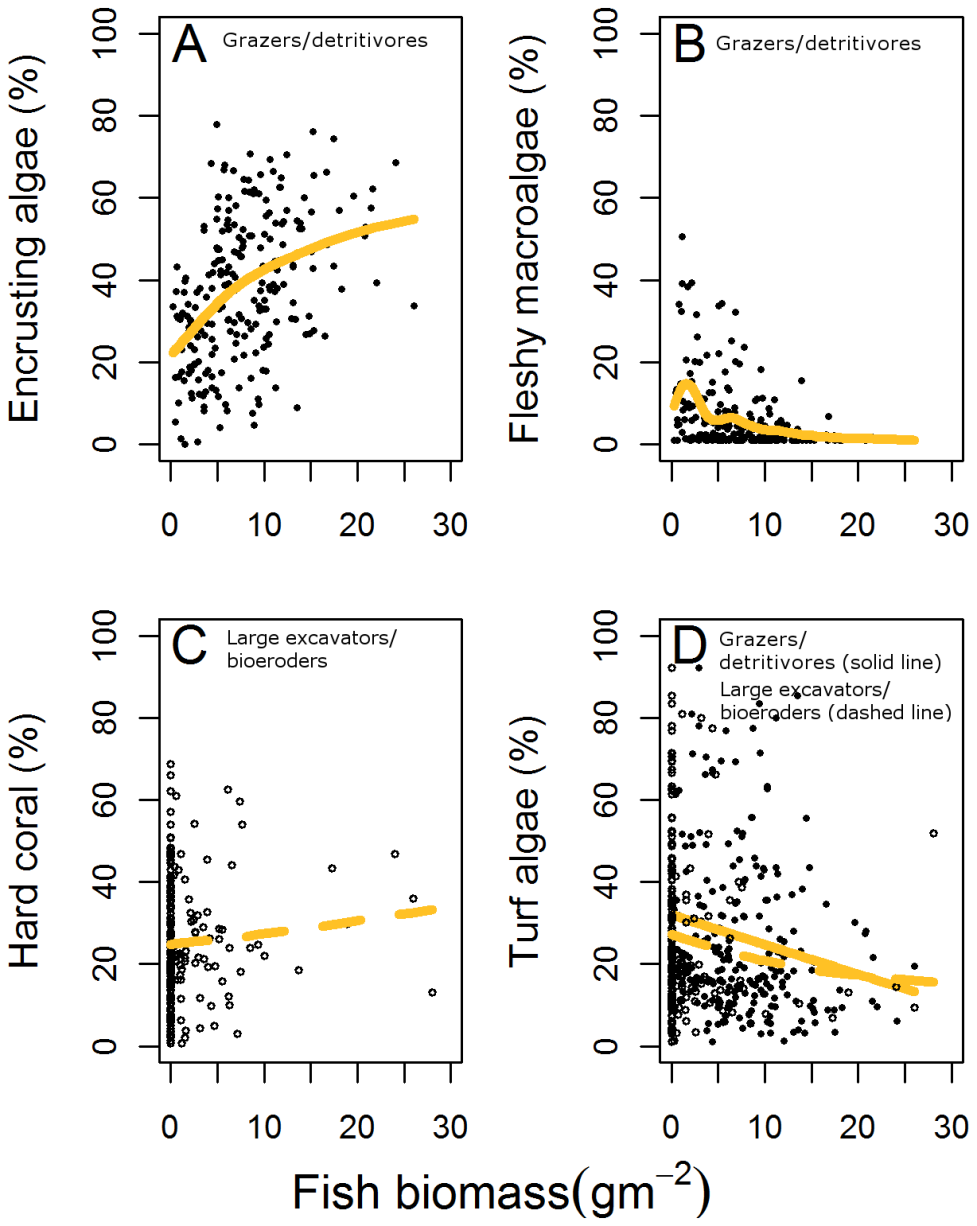
The relationship between the percentage cover of benthic categories with the biomass of herbivorous fish. The best fit relationships identified between (A) encrusting algae and grazers/detritivores (solid points, solid line), (B) fleshy macroalgae and grazers/detritivores (solid points, solid line), (C) hard coral and large excavators/bio-eroders (hollow points, dashed line), and (D) turf algae and grazers/detritivores (solid points, solid line) and large excavators/bioeroders (hollow points, dashed line).

Fleshy macroalgae was the benthic cover type best explained by the biomass of herbivorous fish. Macroalgal cover decreased with increasing biomass of grazers/detritivores (Fig. 4b). The best fitting (lowest AICc, highest weight) models all included grazers/detritivores, while the top candidate model contained the biomass of this single functional group and depth (Table 1, model weight 49% and 50% deviance explained). Models with all herbivores or all fishes as predictor variables had model weights of <0.1% (Table 1).

Similarly, variation in encrusting algal cover was best explained by models which specified herbivorous fish biomass by functional group rather than total herbivore biomass (Table 1, model weight for total herbivore biomass was <0.1%). As with macroalgae, the model with strongest support for variation in encrusting algal cover contained depth and the singular functional group grazers/detritivores (model weight 46%, deviance explained of 42%), with encrusting algal cover decreasing as grazers/detritivore biomass increased (Fig. 4a). All top three models had non-trivial support (weights between 14% and 46%, and deviance explained of 42–43%).

Fits for the other two benthic cover types, hard coral and turf algae, were less clearly improved by breaking herbivores out into different functional groups, as models with all herbivorous fishes or all fishes (herbivores and non-herbivores) had model weights of between 7% and 22%. However, the best models were again those fitted with herbivore functional groups. Specifically, hard coral cover increased with the biomass of large excavators/bio-eroders (Table 1, Fig. 4c). The difference between that model and the alternate top-ranked models was negligible, although models which contained large excavating bio-eroders and excavators tended to have the greatest weight and explained deviance (Table 1). Large excavators/bio-eroders were also amongst the best predictors for turf algal cover, with the best fitting model indicating that turf algae decreased with the biomass of grazer/detritivores and large excavators/bio-eroders (Table 1, Fig. 4d, model weight of 29%, deviance explained of 15%). Although for this benthic category, models with total herbivore biomass and total fish biomass had nearly as much support (model weights of 22% and 19% respectively, and 14–14.5% deviance explained, Table 1).

## Discussion

This study explores the associations between habitat composition and the fish assemblage, specifically, functional groups of herbivorous fish, on the coral reefs of American Samoa. Similar to previous reports from permanent monitoring sites around the island of Tutuila [42], we found the predominant substrate type in the archipelago in hard-bottomed forereef areas to be encrusting algae (∼40%), followed by turf algae (∼25%). Reefs were also characterized by coral cover of ∼25%. In Tutuila, macroalgal cover varies considerably among site locations, but our archipelago-wide estimate of 5% was very similar to what has previously been reported for Tutuila [42].

The presence and abundance of coral reef fishes are often linked to habitat characteristics; for example the abundance of smaller-bodied reef fishes and obligate corallivores, such as butterflyfish, depends heavily on the amount of coral cover [14], [43]. In contrast, herbivorous fishes exhibit considerable variability in distribution and abundance across reef environments of diverse habitat composition as is evident from, e.g., cross-shelf gradients in the functional roles, feeding activity and abundance of herbivore assemblages [22], [44], [45]. Despite the more generalized habitat associations of herbivorous fish, in this study we found benthic cover on coral reefs to be associated with their biomass, and we determined that variability in benthic cover was better predicted by the biomass of herbivores than by total fish biomass (herbivores and non-herbivores combined). Furthermore, for all benthic substrate types assessed (hard coral, fleshy macroalgae, encrusting algae and turf algae), we found benthic cover was better predicted by biomass of particular functional groups of herbivores based on feeding mode (as per [21]) rather than by all herbivorous fishes combined into a single group. This result is consistent with the expectation that herbivores are not equal in terms of their influence on reef benthic composition due to varied feeding rates, preferences and impacts on the benthos [24], [46], [47], [48]. This suggests different groups of herbivores will have diverse long term impacts on benthic community structure, as appears evident from small-scale cage experiments [49]. Our study provides further support for the appreciation that different functional groups of herbivores have varying roles in regulating reef habitat and community structure.

The degree of association between benthic cover and the biomass of different functional groups of herbivores varied considerably by cover type. Fleshy macroalgae was the benthic category most tightly related to the presence of herbivores. This result is consistent with the inverse relationship between herbivorous fish and macroalgal cover [15], [16], [17]. This inverse relationship is further supported by small-scale caged exclusion experiments which have found that fleshy macroalgae proliferate in the absence of herbivores [9], [10], [11], [12], [19] and also by remote video observations on the feeding intensity of herbivores over transplanted patches of macroalgae [48]. High biomass of grazers/detritivores had the strongest association with low cover of both fleshy macroalgae and turf algae. Our findings further support the role that grazers and detritivores play in preventing macroalgae from becoming established by feeding on diminutive algal forms within the turf algal assemblage [9], [19], [47].

Grazers/detritivores, particularly detritivores, are becoming increasingly recognized for their ecological impact on coral reefs [44]. Recent work on the detritivore *Ctenochaetus striatus* has shown that when they brush the epithlic algal matrix for detritus, they also play a significant role in sediment dynamics in addition to ingesting large quantities of turf algae [23], [24]. *Ctenochaetus striatus* was one of the most abundant herbivores in this and previous studies [42], and is common throughout its range in the Indo-Pacific [32], [44], [50]. Our results further emphasize the likely ecological importance of deteritivores – they appeared in 15 of the 23 optimal candidate models identified in our study. Specfically, a high biomass of this group was associated with low cover of fleshy macroalgal and turf algal cover and relatively high cover of encrusting algae and hard coral. Grazers/detritivores could facilitate coral recruitment through the reduction of inhibitive algae and removal of sediment [51], [52].

Grazers/detritivores were the dominant component of herbivorous fish biomass across all depths at all islands, as has been noted previously around Tutuila [42] and on reefs elsewhere in the Pacific (Great Barrier Reef [44], [47]; Guam, Pohnpei and Palau [32]). Notably, we found that a high biomass of this functional group was also associated with high cover of encrusting algae. This result is particularly important, as it indicates a strong positive affliation between grazers/detritivores and reef calcifiers, implying that this functional group may facilitate coral recruitment [53], [54]. However, interpretation of the impact of grazers/detritivores is complicated by the high positive correlation between that group and small scrapers/excavators. We dealt with collinearity between variables by excluding one of the explanatory variables from the analysis [55]. In this case, we opted to exclude scrapers/small excavators as their mean biomass was less than half that of the grazer/detritivore group. A resulting limitation of these findings is an inability to distinguish between the effect these two groups individually may have on benthic cover.

The biomass of large parrotfishes appeared to be positively associated with increased live coral cover. A similar positive yet weak association has been reported between large bodied parrotfishes and coral cover in Micronesia [32]. This weak association may in part be driven by the the fact that excavating parrotfishes can have both positive and negative impacts on coral cover by clearing space for new coral recruits but also by consuming living coral tissue [25], [26]. Corallivory by parrotfishes can lead to a reduction in the cover of some coral species [45] and in some circumstances to a reduction in juvenile coral density [32]. Parrotfish corallivory can be substantial; the giant bumphead parrotfish *Bolbometopon muricatum* can consume an estimated 5 tonnes of reef carbonate per year per individual, over half which is live coral [22]. Therefore where this species is abundant, rates of corallivory can be high. However, the net impact likely depends on the intensity of grazing [10], [56], [57], [58]. Other, potentially important factors not considered in our analyses that are likely to contribute to variation in the live coral cover – large excavators/bioeroders relationship include exposure, reef orientation and structural complexity [32], [42], [59], [60]. Despite these additional sources of variation, our results indicate that on the American Samoan reefs surveyed in this study, large parrotfishes had a net positive association with coral cover.

The data we used for this analysis came from a large-scale survey program that applies a consistent sampling design and visual survey method across 46 individual U.S. islands and atolls across a large area of the Pacific (in an area of 45° latitude and 58° longitude) [61]. With the program's survey method, the stationary point count method, divers aim to record all fish species observed within their cylindrical survey area – a consequence of which is that survey units are relatively small (∼350 m^2^ per site). Relative to more site-attached species, large parrotfish species in the excavating and scraping functional group have a reduced likelihood of being counted in underwater visual survey methods, due to their higher mobility and larger home ranges [41], [62]. Methods that encompass a larger survey area, such as the timed long swim, would likely lead to more frequent encounters with rare and skittish species [63]. The consequences of infrequent encounters are reduced precision, and potentially biased density estimates among functional groups (i.e. some groups under-represented). Therefore, even though we found several strong relationships between the benthos and herbivorous groups, including large excavators/scrapers, it is possible that our results underestimate the strength of some relationships between herbivores and benthic communities. More generally, all visual survey methods have their own strengths and weaknesses, relating to potential impacts of divers' presence on fishes, whether divers are moving and at what speed, divers experience level and training, and whether and what type of transect line is laid out [64], [65], [66]. It is, therefore, not clear what effect, if any, using a different visual survey method might have had on our results. The data used has the advantages of consistency in design, method and personnel, and was available for 240 sites spread across the region, and as several of the significant results indicate, clearly has utility.

Our estimates of herbivore biomass are low relative to other studies from the Pacific. Total herbivore biomass in this study ranged from 3–18 g m^2^, compared to estimates ranging from approximately 3–600 g m^2^, reported outside and inside marine reserves in Fiji [67], and approximately 10–32 g m^2^ reported from the Great Barrier Reef [18]. Whether the lower biomass estimates we report here are an artifact of the sampling design, study scale, or survey method, or whether instead they represent true regional differences in the herbivorous fish assemblage warrants further investigation. In the case of browsers, the biomass of this functional group of macro-algal feeders was not the strongest predictor for macroalgal cover. The biomass estimates reported here are broadly consistent with earlier data gathered by the monitoring program that was collected using the belt transect method (unpublished data). Notably, few browsers e.g. rabbitfish and batfish were recorded, with the implication being that when using diver dependent monitoring data, grazer/detritivore biomass may serve as a more reliable indicator of the ability of a reef to withstand an algal phase shift.

It has been proposed that functional groups of herbivorous fishes may be used as indicators of coral reef resilience to disturbance events [68]. Whether functional classes or groups of herbivores really do have utility as indicators of coral reef resilience depends on whether the relative density of these fishes provides useful information about ecosystem state and associated ecological processes. An implicit assumption in this approach is that the abundance or biomass of functional groups is related to the impact their feeding has on particular types of algae and, in turn, coral-algal dynamic processes. Importantly, our study demonstrates significant associations between the prevalence of specific functional groups of herbivores (measured by biomass) and benthic habitat composition. Furthermore, the relationship between herbivore functional groups and benthic cover were largely as predicted based on the different groups of fishes' feeding modes and feeding preferences. By applying the functional group approach to data collected by an existing monitoring program, our results support the general application of these groups of herbivores as indicators of one aspect of reef resilience, the ability to maintain and/or recover to a coral-dominated state following disturbance.

This study supports the expectation that higher biomass of specific functional groups of herbivorous fishes will generally be associated with lower levels of turf and fleshy macroalgal cover and higher cover of reef calcifiers. We also found positive, although rather weak, evidence to support the theory that large parrotfishes facilitate or sustain higher coral cover [21]. The total area of reef surveyed here (∼85,000 m^2^) was 1.5 to 26 times the area of previous studies and sites were generally much more widely distributed than those surveyed to date, with previous studies finding little or no evidence of an association between coral cover and parrotfishes [42], [59], [60], [69], [70], [71]. One important benefit of large scale surveys, like Pacific RAMP, is that they generate the large scale datasets that may be required to detect ecologically important but weak effects. Conversely, relationships, such as those discernible between herbivorous functional groups and benthic cover from large data sets, may not always be evident in data gathered by reef monitoring programs operating on a more localized scale.
